# Impact of Infective Immigrants on COVID-19 Dynamics

**DOI:** 10.3390/mca27010011

**Published:** 2022-01-29

**Authors:** Stéphane Yanick Tchoumi, Herieth Rwezaura, Mamadou Lamine Diagne, Gilberto González-Parra, Jean Tchuenche

**Affiliations:** 1 Department of Mathematics and Computer Sciences, ENSAI, University of Ngaoundéré, Ngaoundéré P.O. Box 455, Cameroon;; 2 Mathematics Department, University of Dar es Salaam, Dar es Salaam P.O. Box 35062, Tanzania;; 3 Département de Mathématiques, UFR des Sciences et Technologies, Université de Thiès, Thiès 967, Senegal;; 4 Department of Mathematics, New Mexico Tech, Socorro, NM 87801, USA; 5 School of Computational and Communication Sciences and Engineering, Nelson Mandela African Institution of Science and Technology, Arusha P.O. Box 447, Tanzania;; 6 School of Computer Science and Applied Mathematics, University of the Witwatersrand, Private Bag 3, Wits, Johannesburg 2050, South Africa

**Keywords:** COVID-19, infected immigrants, vaccination, basic reproduction number

## Abstract

The COVID-19 epidemic is an unprecedented and major social and economic challenge worldwide due to the various restrictions. Inflow of infective immigrants have not been given prominence in several mathematical and epidemiological models. To investigate the impact of imported infection on the number of deaths, cumulative infected and cumulative asymptomatic, we formulate a mathematical model with infective immigrants and considering vaccination. The basic reproduction number of the special case of the model without immigration of infective people is derived. We varied two key factors that affect the transmission of COVID-19, namely the immigration and vaccination rates. In addition, we considered two different SARS-CoV-2 transmissibilities in order to account for new more contagious variants such as Omicron. Numerical simulations using initial conditions approximating the situation in the US when the vaccination program was starting show that increasing the vaccination rate significantly improves the outcomes regarding the number of deaths, cumulative infected and cumulative asymptomatic. Other factors are the natural recovery rates of infected and asymptomatic individuals, the waning rate of the vaccine and the vaccination rate. When the immigration rate is increased significantly, the number of deaths, cumulative infected and cumulative asymptomatic increase. Consequently, accounting for the level of inflow of infective immigrants may help health policy/decision-makers to formulate policies for public health prevention programs, especially with respect to the implementation of the stringent preventive lock down measure.

## 1. Introduction

COVID-19, a disease that spreads mainly through coughing and sneezing (human-to-human contacts) has led to countries worldwide restricting international arrivals to delay the spread of the disease, as well as implementing a set of non-pharmaceutical interventions (NPIs) such as the most restrictive lock downs, face mask mandates in publicly accessible spaces, social/physical distancing, contact tracing, isolation of contacts, quarantining of confirmed cases and closure or limited openings of shops and schools [1,2], while such effective non-therapeutic prevention measures to control the transmission of the disease have targeted several aspects of public life, including closure of non-essential business, restaurants and other entertainment venues, restrictions in sizes of spontaneous gatherings, the cancellation of events, they are likely not sustainable in the long run [1]. Consequently, with prevention fatigue and the availability of an effective vaccine, governments are easing restrictions, with resumption of international travels. As of May 2020, imported cases accounted for fueling the transmission in many countries [3]. The COVID-19 pandemic with its many variant strains is a global public health concern with massive socio-economic burden on communities. COVID-19 virus direct and indirect transmission has rendered the pandemic outlook highly uncertain [4]. As immigration of infective populations from COVID-19 regions has posed risks to local populations [5], the objective of this study is therefore to numerically investigate the impact of infective immigrants and vaccination on the transmission dynamics of COVID-19, while both therapeutic and non-pharmaceutical interventions have proved paramount to the mitigation of the COVID-19 pandemic [6,7]. Herein, we only consider vaccination that has been so effective in the prevention of new infections from the maiden strain, despite the emergence of the new COVID-19 variants [8,9].

Mathematical models have been instrumental in the understanding and control of disease dynamics. Data analysis to evaluate the risk posed by the daily incoming immigration population is an approach to disease prevention [10]. In fact, Li et al. [5] estimated that about 86% of COVID-19 cases were undocumented before travel restrictions were put in place, and this could help explain the lightning-fast spread of this virus around the world. Studies of the impact of infective immigrants on disease epidemiology abound in the literature, see [3,11–14] and the references therein. Russell et al. [3] investigated the effect of internationally imported cases on internal spread of COVID-19, and concluded that travel bans might have little impact on COVID-19 dynamics except in countries with large inflow of travelers and low COVID-19 incidence. Our study differs from theirs in the sense that we explicitly formulate a mathematical model as a system of nonlinear ordinary differential equations, which captures the dynamics of the disease with inflow of infectives and considers vaccination.

Since mathematical models with inflow of immigrants into either the exposed or the infective class do not admit any disease-free equilibrium nor a basic reproduction number, but only an endemic equilibrium, we will not dwell much on the mathematical analysis of the special case when there is no inflow of infectives, but will instead refer savvy readers to the following articles and the references therein [12,13,15–19]. With the availability of effective vaccines, which is accounted for in our model, we compute and graphically depict the number of deaths, cumulative infected, cumulative asymptomatic and cumulative vaccinated. Our result underscore the importance of taking into account the immigration levels in order to mitigate the spread of COVID-19 pandemic in a country, especially with respect to the implementation of the stringent preventive lock down measure.

The structure of this paper is as follows. The proposed dynamical model is formulated in Section 2. The basic reproduction number of the special case of the model without immigration of infectives is derived in Section 3. Numerical simulations performed to support analytical results are presented in Section 4. The conclusion is provided in Section 5.

## 2. The Model

**Figure 1.**
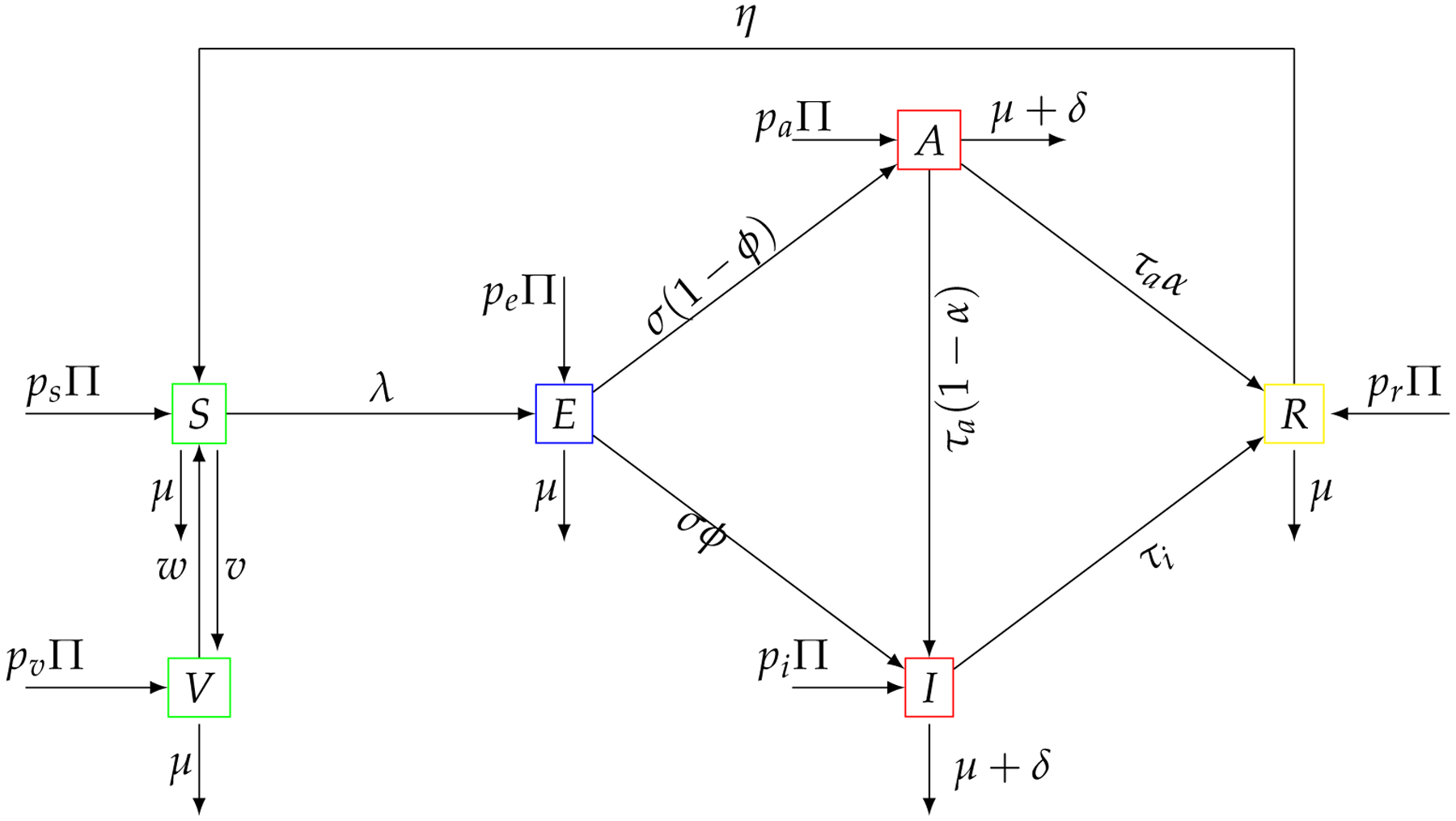
COVID-19 transmission dynamic model flowchart with inflow of infective immigrants.

Consider a homogeneously mixing within the population, i.e., individuals in the population have equal probability of contact with each other. Using a deterministic compartmental modeling approach to describe the disease transmission dynamics, at any time t, the total population N(t) is subdivided into several epidemiological states depending on individuals health status: susceptible S(t), vaccinated V(t), exposed E(t), asymptomatic A(t), infected I(t) and recovered R(t). A compartmental diagram of the deterministic model is given in Figure 1.

From the model flow diagram of the COVID-19 transmission with vaccination and inflow of infective immigrants given in Figure 1, we derive the following system of nonlinear ordinary differential equations:

(1) $$\left\{\begin{array}{l} \frac{dS}{dt}=p_{s}\text{Π}+wV+\eta R-(\lambda +vs.+\mu )S,\\ \frac{dV}{dt}=p_{v}\text{Π}+vS-(w+\mu )V,\\ \frac{dE}{dt}=\lambda S+p_{e}\text{Π}-(\sigma +\mu )E,\\ \frac{dI}{dt}=p_{i}\text{Π}+\sigma \phi E+\tau_{a}(1-\alpha )A-\left(\tau_{i}+\mu +\delta \right)I,\\ \frac{dA}{dt}=p_{a}\text{Π}+\sigma (1-\phi )E-\left(\alpha \tau_{a}+\mu +\delta \right)A,\\ \frac{dR}{dt}=p_{r}Pi+\alpha \tau_{a}A+\tau_{i}I-(\eta +\mu )R, \end{array}\right.$$

where $\lambda =\frac{\beta (\xi A+I)}{N}$. The population size is N=S+V+E+A+I+R, and the initial conditions at time t=0 are S(0)≥0,V(0)≥0,E(0)≥0,I(0)≥0,A(0)≥0,R(0)≥0.

The recruitment rates into the S,V,E and I satisfy the following relation $p_{s}+p_{v}+p_{e}+$ $p_{i}+p_{a}+p_{r}=1$. Table 1 provides all the model parameter values used for the numerical simulations. The values of the parameters were taken from different references related to SARS-CoV-2.

## 3. Model Analysis

As is customary with dynamical models with inflow of infective immigrants, there is no disease-free equilibrium for model (2), as well as no basic reproduction number [12,15–19]. That is, the disease cannot be eliminated unless immigration of infected is halted. Hence, the disease always becomes endemic in the population and tends to a unique globally asymptotically stable endemic equilibrium [18,27].

When $p_{e}=p_{i}=p_{a}=0$, the disease-free equilibrium of the special case of the model (2) with no infective immigrants is given by $E^{0}=\left(S^{0},V^{0},0,0,0,0\right)$, where

$$S^{0}=\frac{\Pi \left[\mu p_{s}+w\left(p_{s}+p_{v}\right)\right]}{\mu \left(\mu +v+w\right)},\text{ and }V^{0}=\frac{\Pi \left[\mu p_{v}+v\left(p_{s}+p_{v}\right)\right]}{\mu \left(\mu +v+w\right)}.$$

To compute the basic reproduction number $R_{0}$, which is defined as the average number of secondary infections generated by a single infectious individual during his entire infectious period in naive population, we use the next generation matrix method in [28]. The associated next generation matrix and the rate of transfer of individual of model (2) are given by

$$\text{ℱ}=\left[\begin{array}{c} \frac{\beta S(\xi A+I)}{N}\\ 0\\ 0 \end{array}\right],\;and\;\nu =\left[\begin{array}{c} g_{1}E\\ -\sigma \Phi E-\tau_{a}(1-\alpha )A+g_{2}I\\ -\sigma (1-\phi )E+g_{3}A \end{array}\right].$$

where $g_{1}=\sigma +\mu ,\;g_{2}=\tau_{i}+\mu +\delta$ and $g_{3}=\alpha \tau_{a}+\mu +\delta$.

Hence, the new infection terms F and the remaining transfer terms V are, respectively, given by

$$F=\left[\begin{array}{ccc} 0 & \frac{\beta }{N^{0}}S^{0} & \frac{\beta \xi }{N^{0}}S^{0}\\ 0 & 0 & 0\\ 0 & 0 & 0 \end{array}\right],and\;V=\left[\begin{array}{ccc} g_{1} & 0 & 0\\ -\sigma \phi & g_{2} & -\tau_{a}(1-\alpha )\\ -\sigma (1-\phi ) & 0 & g_{3} \end{array}\right].$$

Thus,

$$FV^{-1}=\left[\begin{array}{ccc} \frac{\left[(1-\alpha )(1-\phi )\tau_{a}+g_{2}\xi (1-\phi )+g_{3}\phi \right]S^{0}\beta \sigma }{N^{0}g_{1}g_{2}g_{3}} & \frac{\beta S^{0}}{N^{0}g_{2}} & \frac{\left[\tau_{a}(1-\alpha )+g_{2}\xi \right]\beta S^{0}}{N^{0}g_{2}g_{3}}\\ 0 & 0 & 0\\ 0 & 0 & 0 \end{array}\right].$$

The dominant eigenvalue or the spectral radius of the next generation matrix $FV^{-1}$, which represents the basic reproduction number, is given by

(2) $$R_{0}=\frac{\left[\left(1-\alpha \right)\left(1-\phi \right)\tau_{a}+g_{2}\xi \left(1-\phi \right)+g_{3}\phi \right]\left[\mu p_{s}+w\left(p_{s}+p_{v}\right)\right]\sigma \beta }{g_{1}g_{2}g_{3}\left(p_{s}+p_{v}\right)\left(\mu +v+w\right)}.$$

The potential of an epidemic/pandemic to persist or not is based on the value of $R_{0}$ being greater or less than one. The basic reproduction number $R_{0}$, which measures initial disease transmission, is a threshold value that characterizes the local asymptotic stability of the underlying dynamical system [28,29]. Ganasegeran et al. [30] use time-series incidence data to compute the value of the time-dependent reproduction number Rt during the COVID-19 containment measures in Malaysia. They establish that at least 74% of the Malaysian population needed to be vaccinated to achieve herd immunity against COVID-19. A result which is somewhat influenced by the availability of an efficacious vaccine.

**Figure 2.**
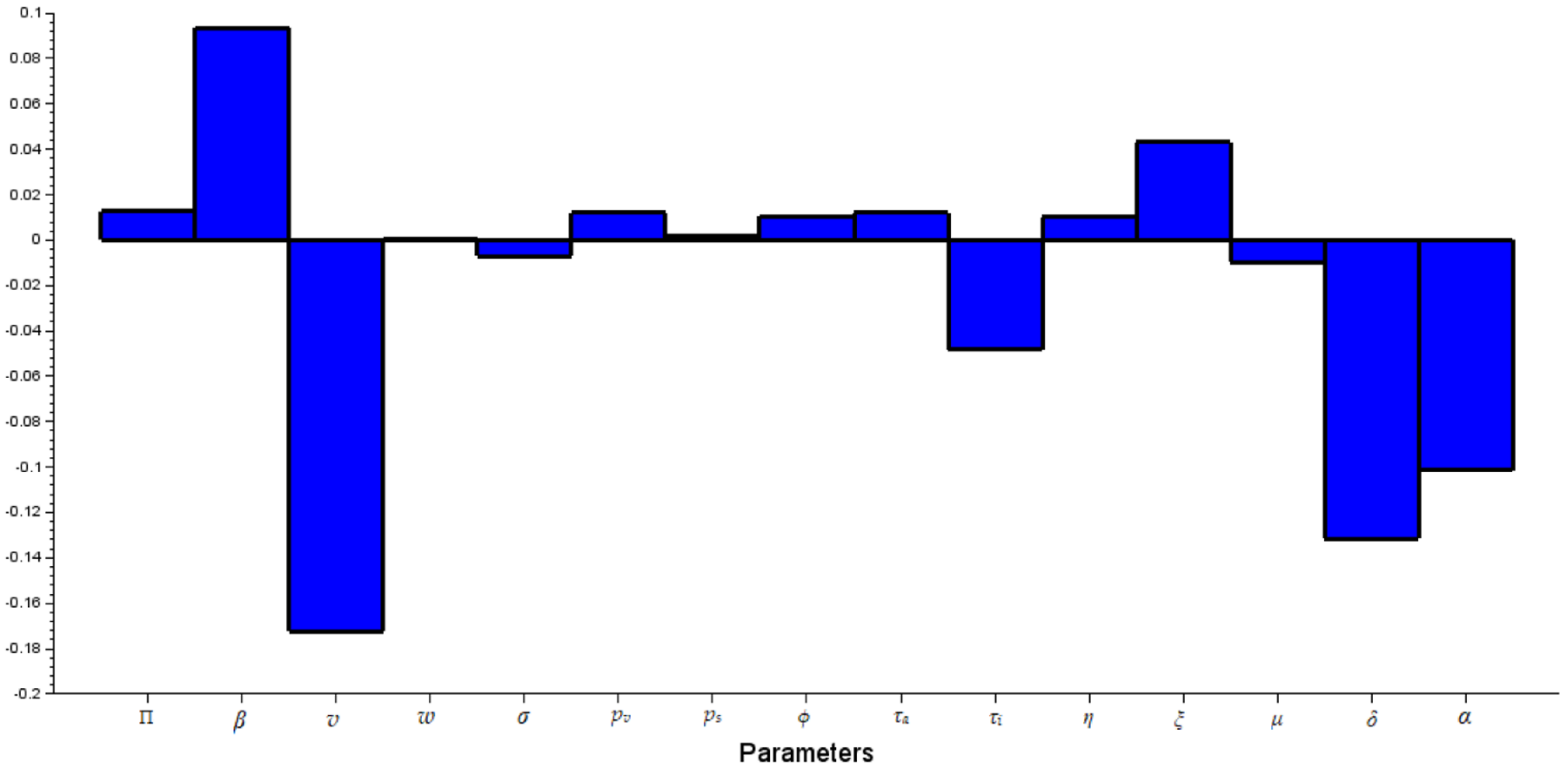
Graphical representation of the sensitivity of the reproduction number $R_{0}$ using Latin hypercube sampling and the Partial rank correlation coefficients with 10,000 samples.

Since by construction symbolic representations of real-life problems could inherit loss of information [31], using the method of Partial rank correlation coefficients (PRCCs) and the Latin hypercube scheme, we perform sensitivity analysis of $R_{0}$ to identify the most sensitive model parameters that significantly impact initial disease transmission. From the results of the sensitivity analysis depicted in Figure 2, we note that the most sensitive parameters to the model when there is no inflow of infectives are: the effective contact rate β, the vaccination rate v, the proportion of asymptomatic who recover naturally α and as expected the disease-induced death rate δ, while the aim of any prevention or therapeutic measure is to avoid/reduce loss of life, the latter suggest that treatment should be given prominence in the fight against the pandemic in order to mitigate the number of deaths.

**Figure 3.**
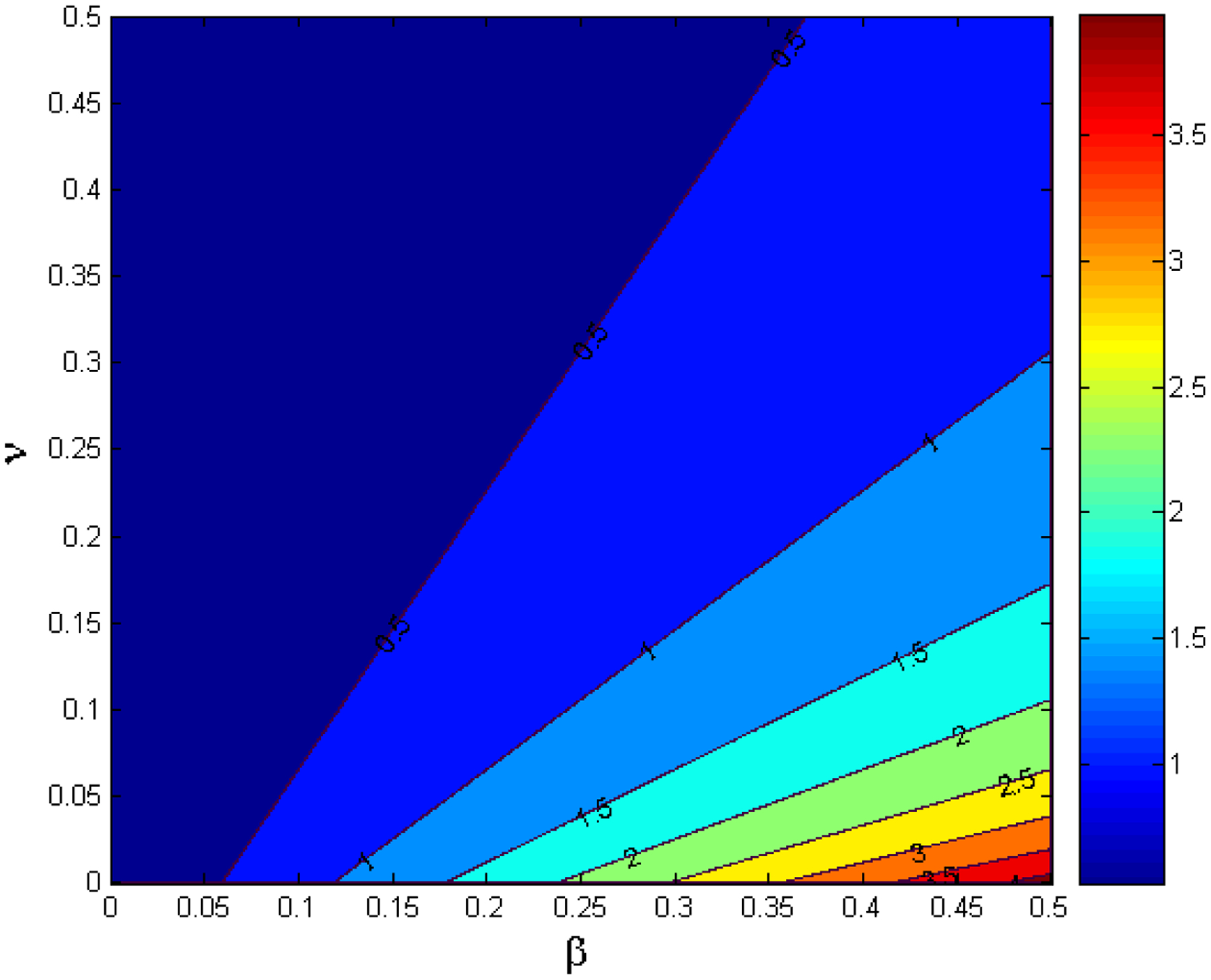
Contour plot of the basic reproduction number $R_{0}$ for different values of the effective contact rate β and the vaccination rate v.

**Figure 4.**
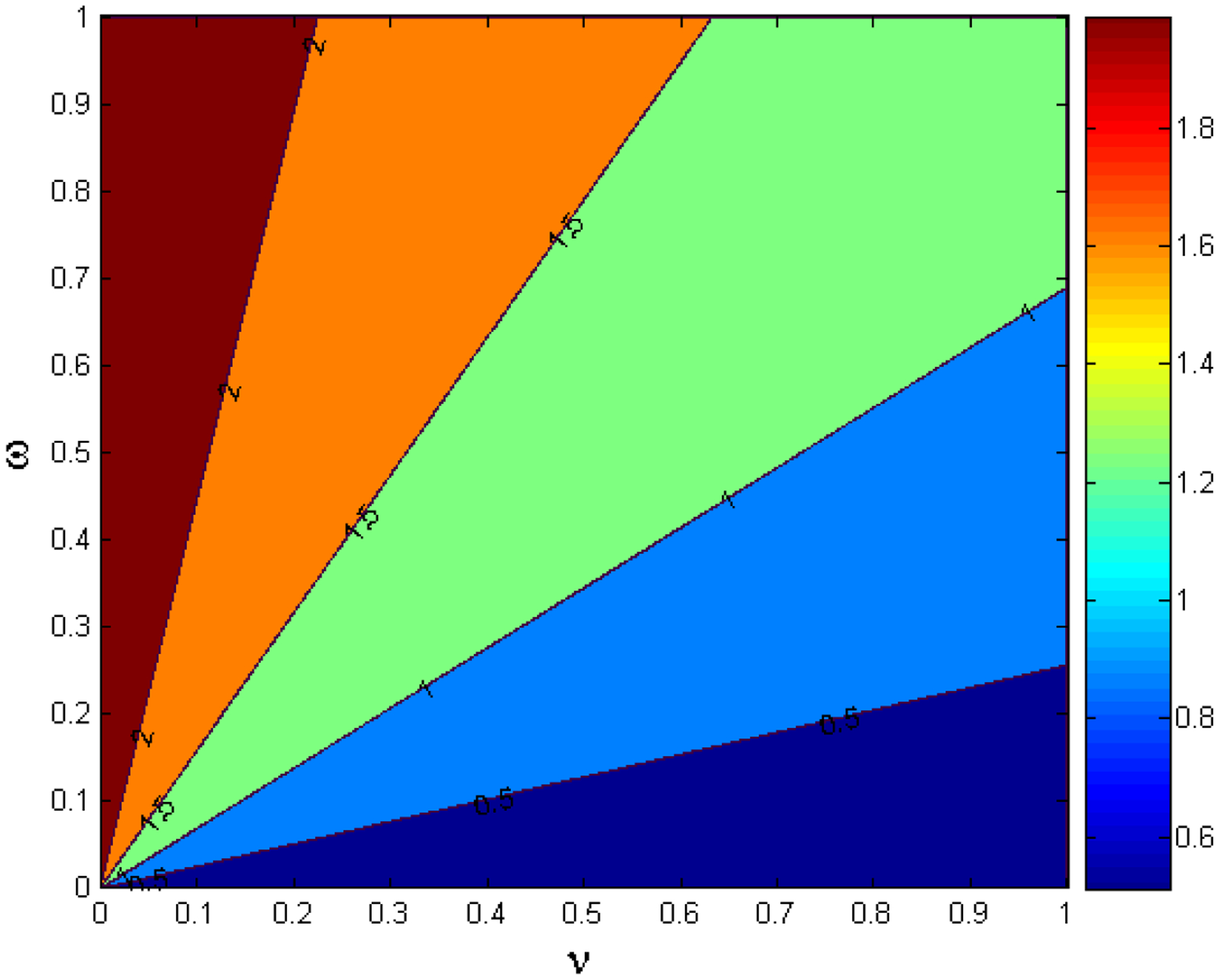
Contour plot of the basic reproduction number $R_{0}$ for different values of the vaccination rate v and waning immunity rate w.

The contour plot of the variations of the transmission and the vaccination rates β and v, respectively, are depicted in Figure 3. As the transmission rate increases, the level of vaccination efforts necessary to control the epidemic also increases, therefore posing a great challenge to the health policy and decision makers. Notice, that for larger values of the transmission rate β larger vaccination rates are needed to bring the basic reproduction number $R_{0}$ below one. Thus, for SARS-CoV-2 variants that are more transmissible and that eventually would become more prevalent in the population, we will need faster vaccination paces in order to reduce $R_{0}$ to less than unity [8,32,33]. Figure 4 depicts the impact of the vaccination waning immunity and the vaccinate rate on the disease dynamics. It can be observed that for larger values of the waning immunity rate ω it is necessary to increase the vaccination rate v in order to be able to reduce the basic reproduction number $R_{0}$. This result is expected since large values of ω translates on people becoming susceptible faster and therefore able to become infective again and then spread SARS-CoV-2.

## 4. Numerical Simulations

Numerical simulations of the model system (2) are preformed by varying some parameter values in order to generate different results regarding the number of deaths, infected and asymptomatic cases. We vary the vaccination and the immigration rates. In addition, we perform additional numerical simulations with a higher infectivity to take into account the scenario when a more transmissible variant such as the current Omicron variant appears. We use the model parameter values and the variable initial conditions given in Table 1. These initial conditions are an approximation of the situation in the US when the vaccination program was starting. We considered data from USA since it is more accurate than in other countries. Therefore, the initial sub-population of vaccinated has been assumed as zero. Slightly varying these initial conditions does not affect the qualitative results that are presented in this section. For all the numerical simulations we use days as the time unit.

### 4.1. Base Scenarios with Lower Transmissibility of the SARS-CoV-2

**Figure 5.**
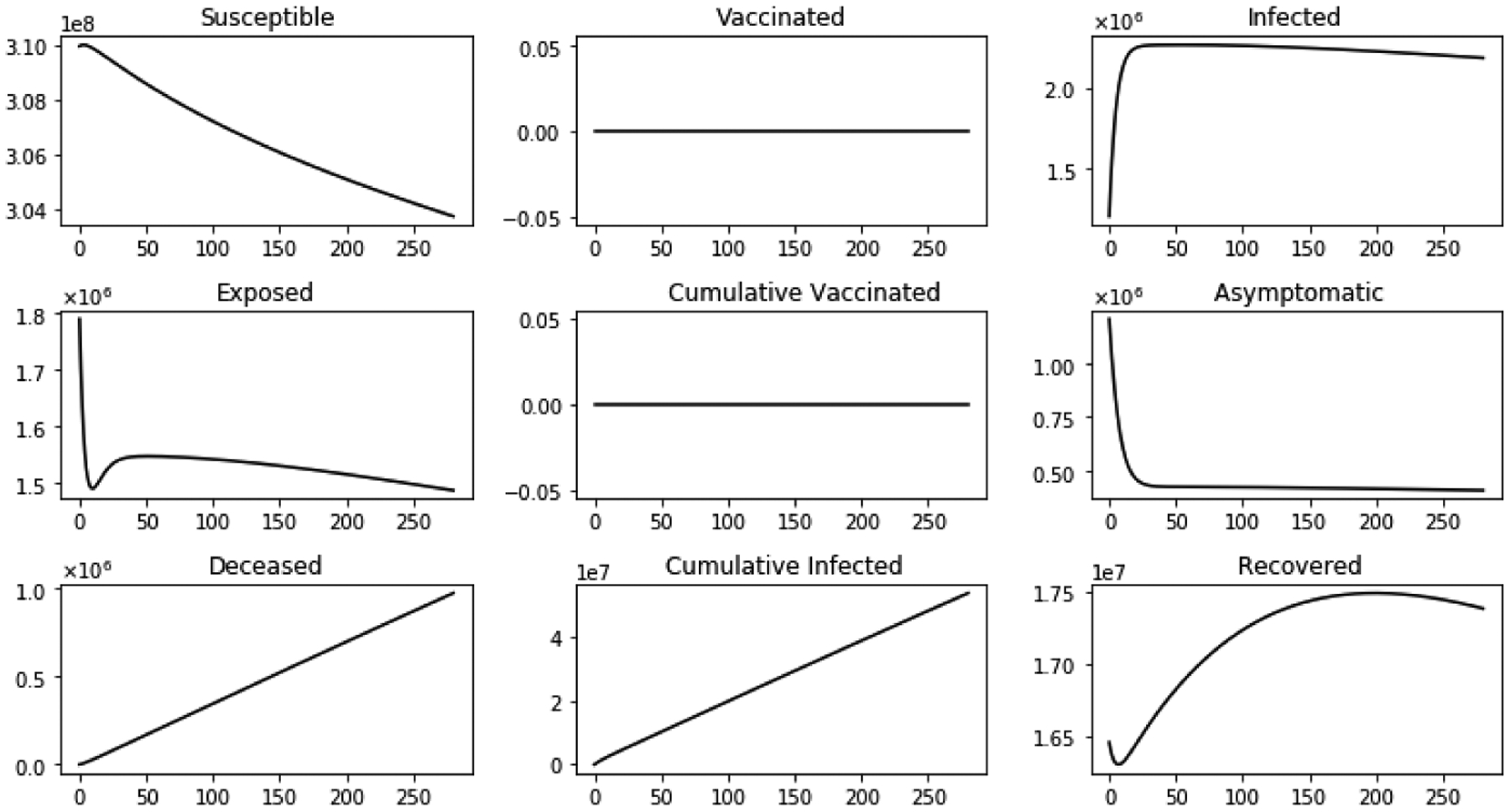
Dynamics of the model sub-populations without inflow of infective immigrants and without vaccination.

The first scenario considered is without immigration in order to compare with the case when immigration is included. Figure 5 shows the dynamics of several sub-populations without immigration and without vaccination. The susceptible sub-population S(t) decreases since the susceptible individuals are infected and transit to the exposed class. The infected sub-population initially increase and reach a peak due to the COVID-19. Later, this sub-population decreases since the susceptible class S(t) decreases significantly, and therefore there are not a great amount of potential candidates to become infected. Exposed sub-population initially decreases since the initial condition is large but later follows the same profile as the infective sub-population. The asymptomatic sub-population decreases despite the infectives increase. This is due to the fact that there are less asymptomatic individuals since some of them become infectives. The vaccinated sub-population V(t) is zero since no vaccination program is considered in this first scenario.

**Figure 6.**
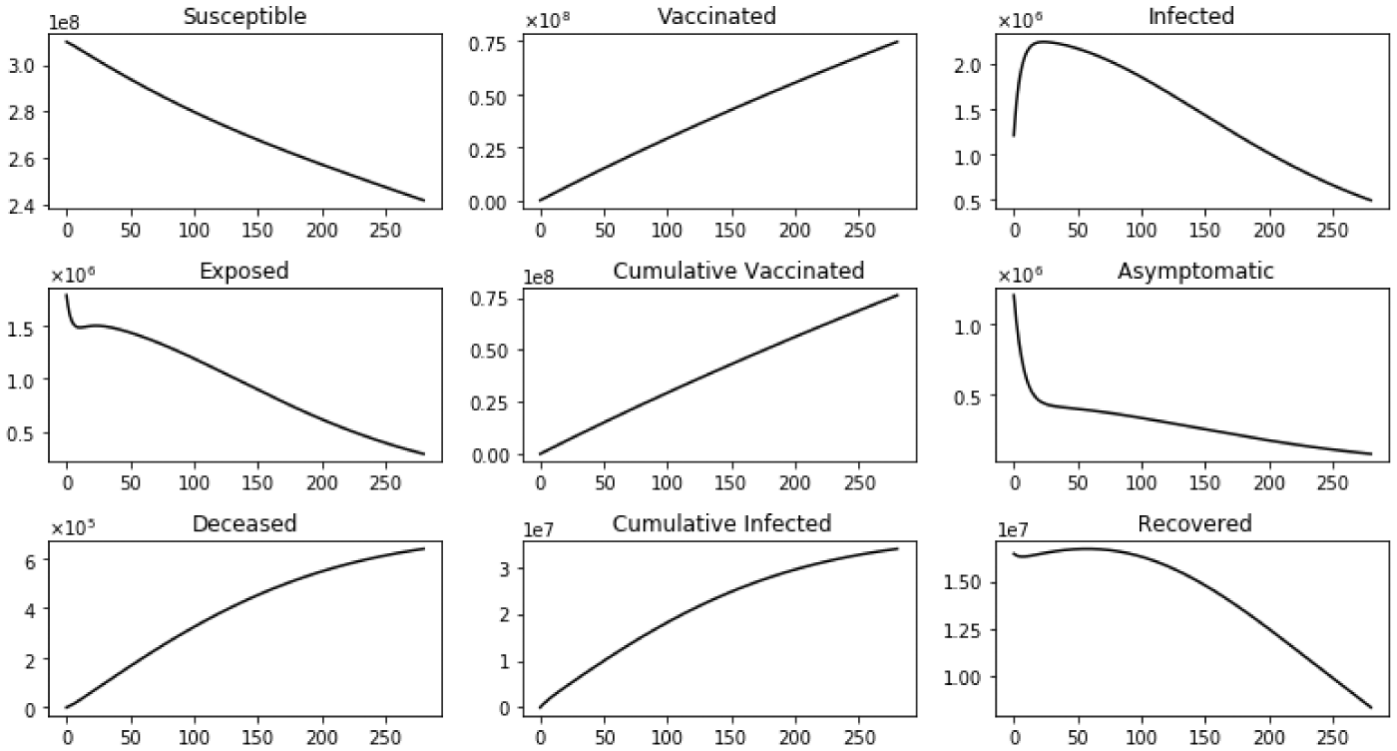
Dynamics of the model sub-populations without inflow of infective immigrants and with vaccination.

The second scenario considered is without immigration and with vaccination. Figure 6 shows the dynamics of several sub-populations. The susceptible sub-population S(t) decreases since the susceptible individuals are infected and also decreases due to vaccinations. Therefore, the vaccinated population increases, especially as we disregard the potential infections from vaccinated individuals. The infected sub-population initially increases due to infections, then reaches a peak and it decreases faster (in comparison with the scenario without vaccination) due to the vaccination program. The asymptomatic sub-population decreases since $\tau_{a}>\tau_{i}$ and ϕ>0.5. Moreover, some of the asymptomatic individuals transit to the infective sub-population. In addition, the exposed and asymptomatic sub-populations decrease.

**Figure 7.**
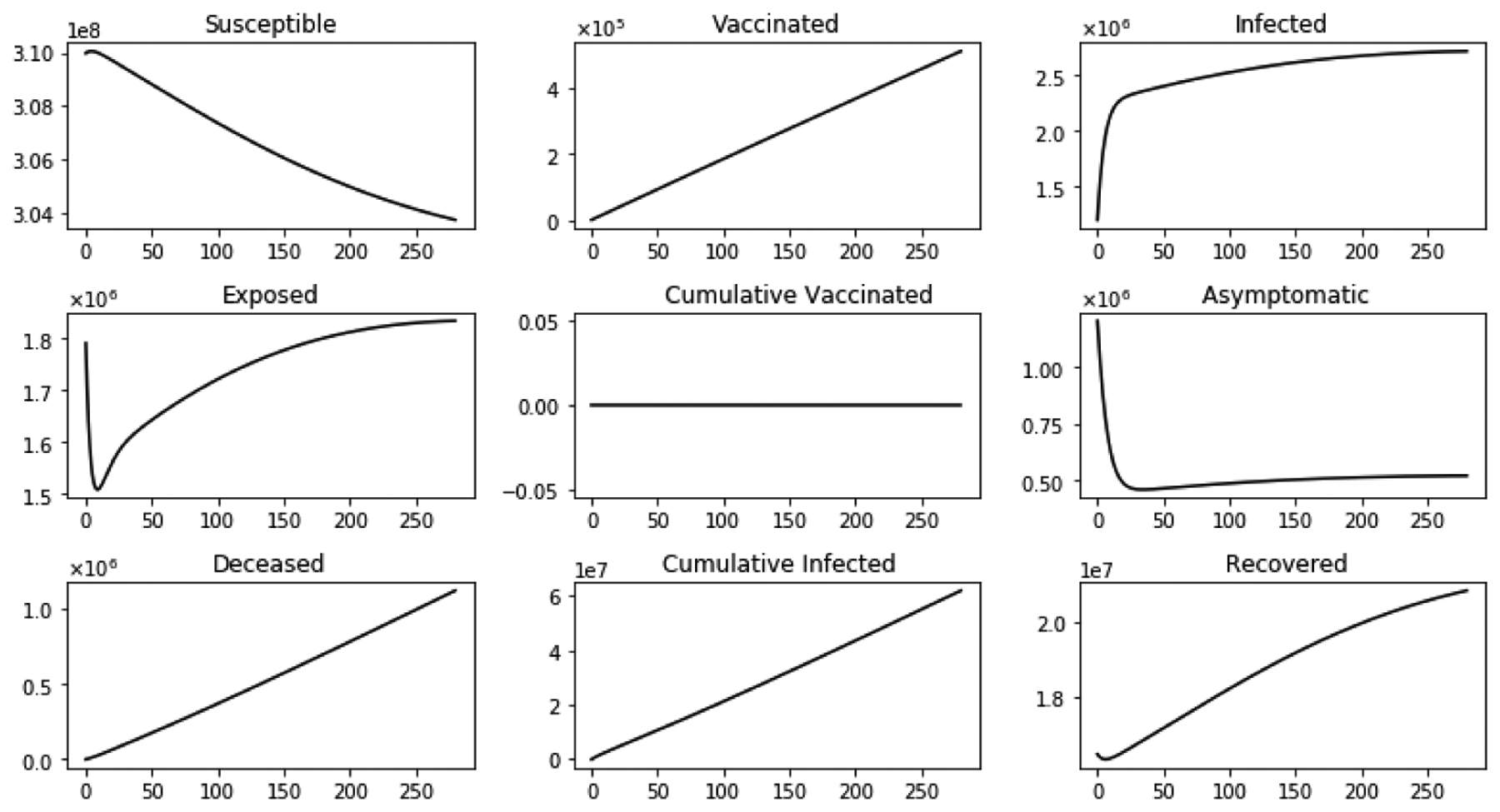
Dynamics of several sub-populations with inflow of infective immigrants and without vaccination.

Figure 7 shows the dynamics of several sub-populations with immigration but without a vaccination program. It can be observed that the susceptible sub-population S(t) decreases and the vaccination sub-population increases due to vaccinated immigrants Again the infected sub-population increases, but the asymptomatic sub-population initially decreases and later on there is a small steady increase. As expected, the cumulative cases, deaths and recovered increase. The disease persist due to a large immigration of infectives and a lack of a vaccination program.

**Figure 8.**
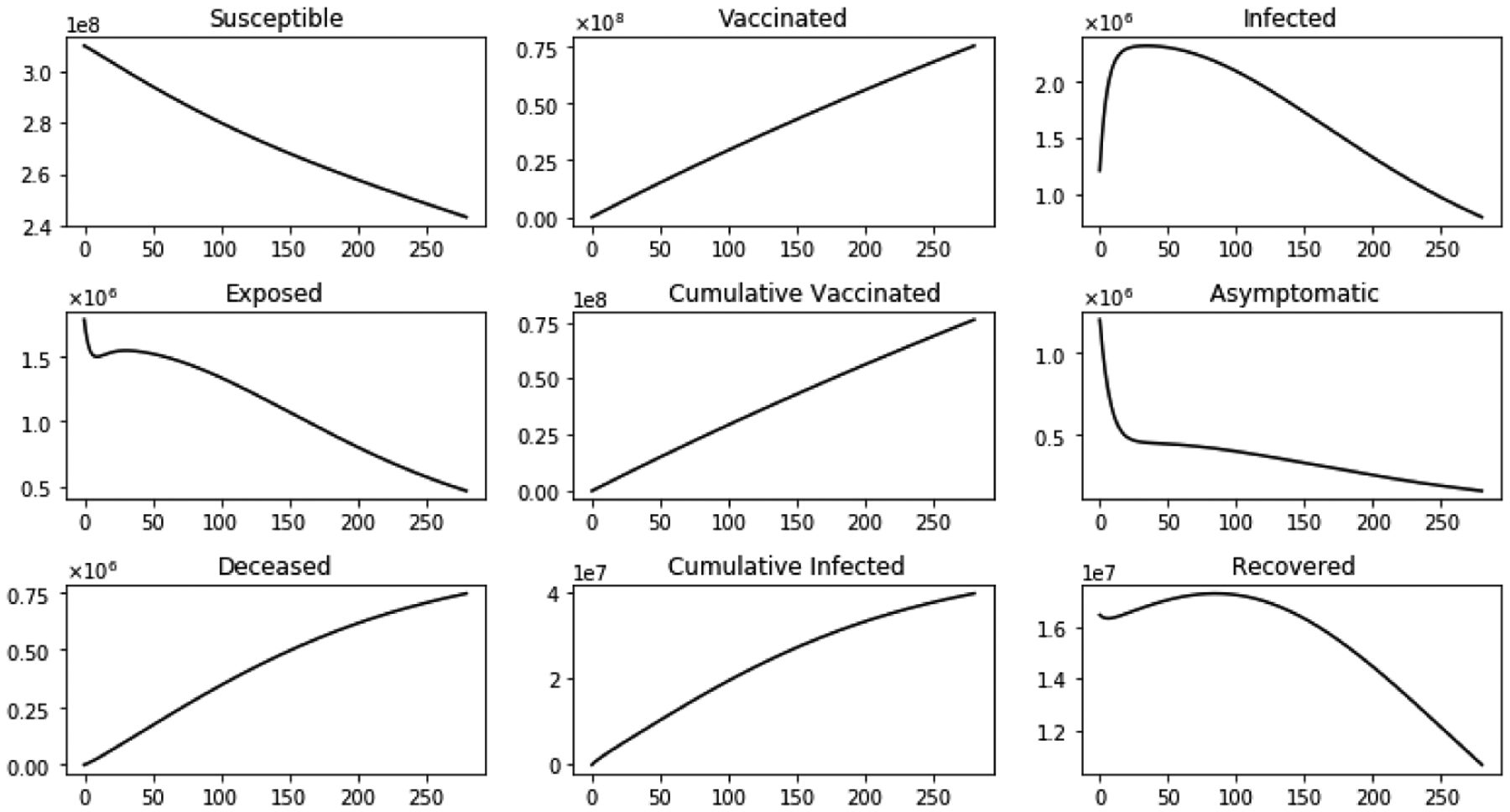
Dynamics of several sub-populations with inflow of infective immigrants and with vaccination.

Figure 8 shows the dynamics of several sub-populations with immigration and with a vaccination program. The infected sub-population I(t) initially increases and then reaches a peak. After the peak, the sub-population decreases since the susceptible sub-population S(t) decreases significantly. The exposed and asymptomatic sub-populations decrease due to the vaccination program and depletion of the susceptible sub-population S(t). Notice that the main difference when immigration is considered is that the number of deaths and cumulative infected are larger than when no immigration is considered. From a health point of view, this means that governments need to take into consideration the immigration in order to avoid additional deaths and hospitalizations. Furthermore, when a vaccination program is implemented, the number of infections and deaths are lower, see Table 2.

### 4.2. Scenario with Higher Transmissibility of the SARS-CoV-2

Here we perform additional numerical simulations with a higher infectivity to take into account the scenario when a more transmissible variant such as the current Omicron variant appears. We use the model parameter values and the variable initial conditions given in Table 1, but we double the transmission rate β and this provides the results presented in Table 3. The table summarizes the results and it can be observed that for a higher contagiousness of the SARS-CoV-2 the number of infected, asymptomatic and deaths increased with comparison when there is a lower SARS-CoV-2 transmissibility. It can be seen that the vaccination reduces the number of infected, asymptomatic and deaths and the incorporation of immigration does the opposite since more infectives are entering the system. However, the immigration becomes less important than when we considered a lower SARS-CoV-2 transmissibility since in this scenario the high transmissibility is enough to spread the disease faster with the initial infective population within the system. In all cases, the vaccination improves the health outcomes related to infective, asymptomatic and deaths. These results are in good agreement with previous studies related to vaccination against SARS-CoV-2 [9,34–36]. With regard to immigration, further studies with real data of immigration is necessary to measure accurately of infective immigrants on the COVID-19 pandemic. Nevertheless, the qualitative results obtained here are worth it to obtain more insight into the complex COVID-19 pandemic.

### 4.3. Further Sensitivity Analysis

**Figure 9.**
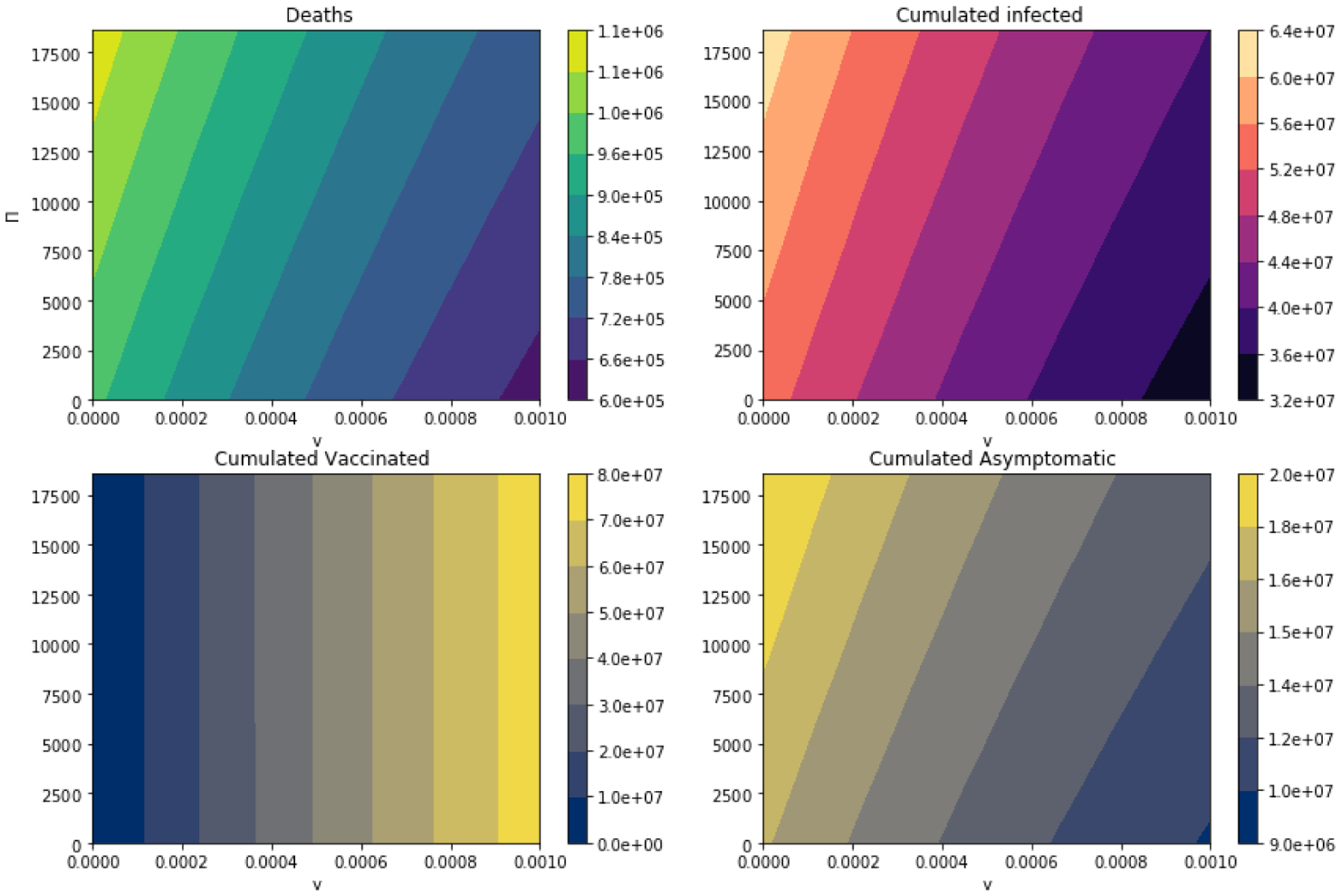
Number of deaths, cumulative infected, cumulative asymptomatic and cumulative vaccinated when vaccination rate and inflow level of immigrants are varied.

We perform additional simulations in order to consider a great variety of scenarios taking into account different vaccination rates and different levels of immigration. In fact we consider infinitely many scenarios from a strict mathematical viewpoint. We compute the number of deaths, cumulative infected, cumulative asymptomatic and cumulative vaccinated. Thus, we can analyze the outcomes under different scenarios. In Figure 9, as expected, increasing the vaccination rate improves significantly the outcomes regarding the number of deaths, cumulative infected and cumulative asymptomatic, see Table 2. On the other hand, when immigration levels are increased, then it can be seen that the situation is worse regarding the number of deaths, cumulative infected and cumulative asymptomatic. Therefore, we can make similar conclusions as above, that is the health authorities (health policy/decision-makers) need to take into account the immigration levels in order to control the COVID-19 pandemic in their respective countries. Lessons from the past two years with multiple lock downs in some countries should also be part of the puzzle especially with respect to the implementation of the stringent preventive lock down measure. In fact, Mugisha et al. [37] noted that even with elimination of all imported cases, mitigating the spread of COVID-19 in Uganda will take almost a year. Therefore, a combination of prevention and therapeutic measures should be implemented to combat the growing threat of this pandemic with its multiple emerging virus strains.

## 5. Conclusions

We formulated a mathematical model to investigate the impact of immigration and vaccination on the number of deaths, cumulative infected and cumulative asymptomatic. We derived the basic reproduction number of the special case of the constructed mathematical model without immigration of infectives. This secondary parameter depends on several factors as expected. As in many epidemic models, one of the main factors is the transmission rate of the virus. Other factor, is the natural recovery rate of infected individuals. In addition, the waning rate of the vaccine and the vaccination rate are also important.

We varied several factors that affect the total number of deaths, cumulative infected and cumulative asymptomatic. These are the immigration levels and vaccination rate. Graphically displayed results from the numerical simulations show that increasing the vaccination rate improves significantly the outcomes regarding the number of deaths, cumulative infected and cumulative asymptomatic. However, when immigration levels are increased, the number of deaths, cumulative infected and cumulative asymptomatic increase significantly. Thus, accounting for the level of inflow of infective immigrants may help health authorities and health policy/decision-makers to formulate policies for public health prevention programs.

Finally, we would like to mention some natural limitations of this work. As with any mathematical model for infectious diseases, there are limitations due to the complexity of the reality. The proposed model does not consider co-existence of SARS-CoV-2 variants. Accurate values for some parameters are still unknowns and this could affect our quantitative results, but the sensitivity analysis suggests that qualitative results would not change. Future studies could consider investigating the impact of infective immigrants when other well know prevention measures and treatment intervention are implemented in addition to the vaccination program considered herein. With multiple variants of the disease, extending the proposed model to include several potential infected classes representing each a different strain of the disease could be viable [38]. For mathematical tractability and convenience, detailed analysis of the special case of the extended model without infective immigrants could be an interesting exercise similar to the work conducted in the [3,11–14], while model extension is great, fitting the model to a country specific data and estimating most of the model parameter values based on the data is important to inform decision making.

## Figures and Tables

**Table 1.** Model parameters and variables.

Parameter	Description	Value	Unity	Reference
Π	Recruitment rate	$\frac{4\times 10^{8}}{59\times 365}$	_day_^−1^	Assumed
*β*	Effective contact rate	0.09	_day_^−1^	Assumed
*ν*	Vaccination rate	0.001	_day_^−1^	Assumed
*ω*	Vaccine waning rate	0.0001	_day_^−1^	Assumed
*σ*	Exit rate from the exposed class	0.13	_day_^−1^	[20]
*α*	Prop. of asymptomatic who recover naturally	0.14	_day_^−1^	[20]
*p*_*s*_ *, p*_*ν*_ *, p*_*e*_ *, p*_*i*_ *, p*_*a*_ *, p*_*r*_	Recruitment prop. into the *S, V, E, I, R* and *A*	variable	percentage	
*Φ*	Prop. of exposed who become infected	0.7	day^−1^	[21]
*τ*_*a*_	Natural recovery rate of asymptomatic	0.13978	day^−1^	[21,22]
*τ*_*i*_	Recovery rate of symptomatic	0.0833	day^−1^	[22]
*η*	Rate at which recovered ind. become suscep.	0.011	day^−1^	[23]
*ξ*	Reduction in transmission from asymptomatic	0.3	1	[21]
*μ*	Natural mortality rate	$\frac{1}{59\times 365}$	day^−1^	[24–26]
*δ*	Disease-induced death rate	0.018/12	_day_^−1^	Assumed
Variables	Description	Initial Value at *t* = 0		
*S*	Susceptible	309,974,354		
*V*	Vaccinated	0		
*E*	Exposed	1,788,800		
*A*	Asymptomatic	1,204,000		
*I*	Infected	1,204,000		
*R*	Recovered	16,462,937		
*N*	Total population	330,705,643		

**Table 2.** Impact of immigration and vaccination on the cumulative infected, cumulative asymptomatic and deaths.

Immigration	Vaccination	Infected	Asymptomatic	Deaths
No	No	5.372 × 10^7^	1.666 × 10^7^	9.731 × 10^5^
No	Yes	3.393 × 10^7^	1.036 × 10^7^	6.392 × 10^5^
Yes	No	6.194 × 10^7^	1.949 × 10^7^	1.115 × 10^6^
Yes	Yes	3.991 × 10^7^	1.247 × 10^7^	7.440 × 10^5^

**Table 3.** Impact of immigration and vaccination on the cumulative infected, cumulative asymptomatic and deaths. The SARS-CoV-2 transmission rate considered here is β=0.18.

Immigration	Vaccination	Infected	Asymptomatic	Deaths
No	No	4.467 × 10^8^	1.407 × 10^8^	8.003 × 10^6^
No	Yes	3.669 × 10^8^	1.154 × 10^8^	6.621 × 10^6^
Yes	No	4.518 × 10^8^	1.425 × 10^8^	8.090 × 10^6^
Yes	Yes	3.719 × 10^8^	1.172 × 10^8^	6.709 × 10^6^

## Data Availability

Data are contained within the article.

## Abbreviations

The following abbreviations are used in this manuscript:

COVID-19: Coronavirus disease of 2019
